# The Grenada Learning and Memory Scale: Psychometric features and normative data in Caribbean preschool children

**DOI:** 10.1017/S1355617724000481

**Published:** 2024-10-01

**Authors:** Karen Blackmon, Roberta Evans, Lauren Mohammed, Kemi S. Burgen, Erin Ingraham, Bianca Punch, Rashida Isaac, Toni Murray, Jesma Noel, Cora Belmar-Roberts, Randall Waechter, Barbara Landon

**Affiliations:** 1 Department of Psychiatry, Dartmouth Geisel School of Medicine, Hanover, NH, USA; 2 Brain and Mind Institute, Aga Khan University, Nairobi, Kenya; 3 Caribbean Center for Child Neurodevelopment at Windward Islands Research and Education Foundation, Grenada, West Indies; 4 Department of Educational Services, St. George’s University, Grenada, West Indies; 5 Sandilands Rehabilitation Center, Nassau, Bahamas; 6 Department of Environmental and Global Health, Gainesville, FL, USA

**Keywords:** Global health, sustainable development goals, child development, neuropsychology, memory, psychometrics

## Abstract

**Objective:**

Neuropsychological assessment of preschool children is essential for early detection of delays and referral for intervention prior to school entry. This is especially pertinent in low- and middle-income countries (LMICs), which are disproportionately impacted by micronutrient deficiencies and teratogenic exposures. The Grenada Learning and Memory Scale (GLAMS) was created for use in limited resource settings and includes a shopping list and face-name association test. Here, we present psychometric and normative data for the GLAMS in a Grenadian preschool sample.

**Methods:**

Typically developing children between 36 and 72 months of age, primarily English speaking, were recruited from public preschools in Grenada. Trained Early Childhood Assessors administered the GLAMS and NEPSY-II in schools, homes, and clinics. GLAMS score distributions, reliability, and convergent/divergent validity against NEPSY-II were evaluated.

**Results:**

The sample consisted of 400 children (190 males, 210 females). GLAMS internal consistency, inter-rater agreement, and test-retest reliability were acceptable. Principal components analysis revealed two latent factors, aligned with expected verbal/visual memory constructs. A female advantage was observed in verbal memory. Moderate age effects were observed on list learning/recall and small age effects on face-name learning/recall. All GLAMS subtests were correlated with NEPSY-II Sentence Repetition, supporting convergent validity with a measure of verbal working memory.

**Conclusions:**

The GLAMS is a psychometrically sound measure of learning and memory in Grenadian preschool children. Further adaptation and scale-up to global LMICs are recommended.

## Introduction

Assessment of early child neurodevelopment is essential for monitoring brain health at the individual and population levels. At the individual level, timely neurodevelopmental assessment can provide insight into risk for later academic challenges, recommendations for interventions, and progress post-intervention (Waechter et al., 2022). At the population level, accurate neuropsychological surveillance contributes to risk modeling (World Health Organization, 2024), understanding neurodevelopmental disease burden (Arora et al., 2018; Bitta et al., 2017), and evaluating responses to policy changes and public health initiatives (Petrowski et al., 2023).

Pediatric brain health surveillance needs to be multidimensional to capture the range of functions that rapidly mature in the early years. Learning and memory are key components; however, there are currently limited measures that specifically probe the emerging episodic memory system in preschool children (Baron et al., 2014), particularly in limited resource settings (Semrud-Clikeman et al., 2016). Most neurodevelopmental packages indirectly assess memory in young children via measures of attention, working memory, and language (Korkman et al., 2007), despite evidence of rudimentary episodic memory skills as early as 3 years of age (Hayne & Imuta, 2011). This limits the detection of early vulnerabilities in the developing memory system that may be a harbinger of later learning problems.

During preschool age (3–5 years), there is rapid development of hippocampal-cortical memory systems that serve as the substrates for lifelong learning (Bethlehem et al., 2022). While 3-year-old children may struggle to retain newly learned material after long delays, 4-year-olds demonstrate the ability to retain material over a week (Scarf et al., 2011). The maturation of these systems is sensitive to the interaction between genetics, epigenetics, and environmental stressors (Boivin et al., 2015). Stressors include malnutrition (Abubakar et al., 2010; Razzaq et al., 2023), poverty (Noble et al., 2015), adverse experiences (Hughes et al., 2017), and infections (Laughton et al., 2013), all contributing to a high burden of neurodevelopmental disorders in low- and middle-income countries (LMICs) (Bitta et al., 2017). Yet, most existing pediatric neuropsychological measures were developed in high-income countries (HICs) and are often deployed with minimal adaptation, validation, or normative sample development in LMICs (Semrud-Clikeman et al., 2016). This contributes to measurement imprecision in regions where accurate data capture is needed the most, due to disproportionately higher risk exposures.

As of now, there are no existing measures of pediatric learning and memory that have been developed and validated for use in the English-speaking Caribbean. To fill this gap, we collaboratively developed a novel pediatric measure of learning and memory in partnership with community stakeholders in Grenada, West Indies. Here, we describe the Grenada Learning and Memory Scale (GLAMS) and present its psychometric and normative properties in a large sample of preschool children. We hypothesize that the GLAMS will show acceptable reliability and internal consistency, as well as a positive association with age and NEPSY-II subtests. This would provide preliminary evidence for the GLAMS as a valid measure of preschool learning and memory in the English-speaking Caribbean.

## Methods

### Ethical considerations

This study was approved by the St George’s University Institutional Review Board in Grenada (IRB #14099 and #22030) and conducted in accordance with the Helsinki Declaration. Primary caregivers provided written consent for children in their care to participate in the study.

### GLAMS development

The development team consisted of two Caribbean community and clinical psychologists (EI, KSB), one Caribbean public health investigator (BP), one Grenadian visual artist (RI), and two American clinical neuropsychologists (BL, KB). Our objective was to create a measure with the following components: (1) immediate learning, delayed recall, and recognition, (2) auditory-verbal and visual-verbal associative memory, (3) parametric increase in difficulty level to account for steep development/maturation curves in the 3- to 5-year age range, (4) procedures and stimuli familiar to children in the region, (5) short administration time, and (6) easy-to-use and inexpensive materials that could be disseminated at low cost in limited resource settings.

After an iterative process that involved trialing of various instruction sets, paradigms, stimuli, and difficulty levels in focus groups consisting of community stakeholder groups from Grenada (students, nurses, teachers, and social workers), as well as pilot testing and interviews with expert mental health providers, the final version of the GLAMS included two subtests: a verbal list learning and recall task and a face-name associative learning and recall task. Two versions were developed: a T4 version for 3-year-olds, featuring four target items to learn in each task, and a more challenging T6 version for 4- to 5-year-olds, with six target items.

### Shopping List Test (SLT)

This auditory-verbal list-learning test simulates a trip to the store, where the child assists the administrator in remembering items to buy – a scenario endorsed by community stakeholders as a common event for young children. This paradigm was adapted from the California Verbal Learning Test – Children’s Version (CVLT–C) (Delis et al., 1994), designed to measure memory in children from 5 to 16 years of age, and NEPSY-II’s “List Memory” subtest (Korkman et al., 2007), which assesses memory in children from 7 to 16 years of age. Shopping List Test (SLT) items were selected based on cultural familiarity. Both the T4 and T6 versions include three learning trials, a delayed recall trial, and a recognition trial.

- *SLT Total Learning*: In the three learning trials, the administrator reads the list of items aloud at a rate of one word per second and immediately prompts the child to recall the items in any order. The total learning score is the sum of correctly recalled items across the three trials, which ranges from 1 to 12 in the T4 version and from 1 to 18 in the T6 version, with higher scores indicating better learning performance.
- *SLT Delayed Recall*: After a 20-minute distraction-filled delay interval, the child is asked to recall items from the shopping list. The delayed recall score is the total number of correctly recalled items and ranges from 0 to 4 in the T4 version and 0 to 6 in the T6 version.
- *SLT Recognition:* During the recognition trial, the child is read a list of items and asked to respond “yes” or “no” based on whether each item was on the shopping list. In the T4 version, the recognition trial list contains the four target items, four semantically similar foils, and four semantically unrelated foils, sequenced in random order. The T6 version includes six target items, six semantically similar foils, and six semantically unrelated foils, also sequenced randomly. For both the T4 and T6 versions, correct endorsement of target items earns 1.5 points credit, a half-point deduction is made for incorrect endorsement of semantically related foils, and a full-point deduction is made for incorrect endorsement of semantically unrelated foils. Scores range from −6 to 6 for the T4 version and −9 to 9 for the T6 version. Higher scores indicate better signal-to-noise discrimination, zero scores suggest a positive response bias (saying “yes” to every word), and negative scores suggest an atypical endorsement style, possibly indicative of guessing or low engagement.

### Face-Name Binding Test (FNBT)

The Face-Name Binding Test (FNBT) subtest involves associating 2D drawings of children’s faces with names. Adapted from the NEPSY-II’s “Memory for Names” subtest (Korkman et al., 2007), the test features sketches of children’s faces created by the visual artist on the GLAMS development team. The faces of children were designed to resemble Grenadian children, the large majority of whom are of Afro-Caribbean descent. Additionally, names commonly used in Grenada were selected by study team members.

- *FNBT Learning*: The administrator presents each stimulus (child’s face) for 5 seconds at the child’s eye level, while stating their name. The child is then prompted to repeat each name within the 5-second exposure before proceeding to the next item. The first recall trial begins with the administrator showing each face again and asking the child to recall the associated name. If the child responds correctly, the administrator acknowledges the correct answer and then repeats the response. If the child responds incorrectly or does not respond after 5 seconds, the administrator provides the correct name and asks the child to repeat it before being shown the next face. This process is repeated for the second and third trials. The total learning score is the sum of names correctly recalled across three learning trials, ranging from 1 to 12 in the T4 version and 1 to 18 in the T6 version.
- *FNBT Delayed Recall:* After a 20-minute delay interval, the child is shown the faces again and asked to recall the corresponding names. The delayed recall score is the total number of names correctly recalled, ranging from 0 to 4 for the T4 version and 0 to 6 for the T6 version. Higher scores reflect better performance.

### Participants

Children aged 3 and 5 years, whose primary language is English, were eligible for participation. Exclusion criteria included sensorimotor issues that might impede testing, a history of neurological disease/illness/injury, and a clinical diagnosis of a developmental delay (based on parental report). In the first recruitment wave (cohort 1), participants were recruited from 24 preschools across Grenada. Study details were explained to parents and primary guardians (referred to as “caregivers”) by research personnel at parent–teacher meetings. Caregivers received copies of the informed consent form and the study sociodemographic and medical history questionnaire. They were given the opportunity to review the forms, ask questions, and take the forms home. Parents who returned the signed consent form and questionnaire, and whose children met the study’s inclusion/exclusion criteria, were enrolled. A second wave of recruitment was initiated for the assessment of test-retest and inter-rater reliability (cohort 2). During this wave, children were recruited through existing community partnership networks across Grenada. Study team members contacted parents, teachers, and social intervention community workers to explain the study and seek assistance in recruiting parents/children. Parents of children in cohort 2 were asked to provide consent to video record their child’s assessment, intended for evaluating inter-rater reliability.

### Sociodemographic and medical history questionnaire

The sociodemographic and medical history form gathered information about the child’s age (in months), sex, and any existing medical concerns, as well as their primary caregiver’s education level (primary, secondary, tertiary, or above), income bracket (monthly income <500; 500–1000; 1001–2000; 2001–3000; or >3000 Eastern Caribbean dollars), and marital status (married, widowed, divorced, separated, domestic relationship, or single/never married).

### Neuropsychological test battery

Children in both cohorts completed the same test battery, which included the GLAMS and selected subtests from the NEPSY-II. A team of four Trained Early Childhood Assessors (TECAs), trained by neuropsychologists (BL, KB), administered the tests in accordance with standardized procedures. The TECAs were also trained to observe children’s behavior for signs of fatigue, disengagement, frustration, and distractibility and to intervene with attempts to recapture attention, eliminate distractions, downregulate frustration, or reschedule the assessment if necessary. The training was completed before the study onset and refreshed throughout the study duration. The specific NEPSY-II subtests and administration order are detailed in Table 1. Test order was consistent across cohorts. The assessment duration was 1 hour.

### Setting

For cohort 1, assessments were conducted in schools, homes, and occasionally public places. In school settings, the context varied based on availability. Testing was sometimes completed in large rooms separate from the classrooms, providing a quiet and focused environment. In other instances, assessments occurred in multipurpose rooms, schoolyards, or on porches just outside the classrooms, leading to occasional interruptions and distractions, especially during snack or lunch breaks. Due to the COVID-19 pandemic, schools were closed between September 2020 and April 2021. During this period, assessments took place in the participants’ homes with safety and exposure protocols in place, including sanitation, ventilation, and masking (TECAs wore masks). The prevalence of multigenerational households meant that other people in the home were occasionally a source of distraction. As COVID-19 restrictions eased, testing environments varied from parents’ workplaces to public parks. Assessors were trained to manage the testing environment to ensure children could focus on tasks. However, it is important to note that data were often collected in non-standardized environments with multiple distractions and interruptions.

In cohort 2, children were randomly selected to complete the assessment in their home, school, or local health clinic, while adhering to pandemic protocols. The assessment battery was administered on two separate occasions, 14 days apart, in the same setting and with the same TECA, to assess test-retest reliability. Video recordings were obtained from a subsample of 12 children to evaluate inter-rater agreement across four raters.

### Statistical analysis

All data were cleaned in Microsoft Excel and analyzed using IBM SPSS v.29. To enhance interpretability, scores of zero were excluded from the total learning and corresponding delayed recall trials prior to analyses. This was due to uncertainty about whether a zero-learning score reflects absent engagement or genuinely low performance in preschool children. GLAMS subtest score distributions were explored for normality, skewness, and kurtosis using descriptive statistics. Correlational analyses and mean comparisons were used to explore associations between GLAMS subtest scores and sociodemographic data, with test selection informed by whether assumptions of normality were met. Measures of effect size included Cohen’s *d* for mean comparisons and Cramer’s *V* for associations between categorical variables. We classified learning curves as “zero learning,” “some learning,” or “positive learning” and assessed these categories across each age bracket (6-month increments). We used Cronbach’s alpha to evaluate internal consistency across and within GLAMS subtests and exploratory factor analysis to probe the latent factor structure of the five main GLAMS subtests. NEPSY-II subtest scores were used to assess construct-related validity. Specifically, we used the NEPSY-II Sentence Repetition to assess convergent validity with a gold standard preschool working memory test and the visuomotor precision subtest to assess divergence from the presumed orthogonal construct of visuomotor speed and dexterity. Test-retest correlations were assessed with Pearson’s *r* and interpretations adhered to consensus standards of acceptable (>0.60), good (0.70–0.79), very good (0.80–0.89), and excellent (≥0.90) reliability. Fleiss’ kappa was used to assess inter-rater agreement across four independent raters. Qualitative labeling of agreement was derived from standard effect size reporting (Cohen, 1988).

## Results

### Sample demographics

#### Cohort 1

A total of 311 primary caregivers provided consent and completed the sociodemographic/medical history form. Seven children were excluded based on neurological disease/illness/injury. Caregivers endorsed mild medical issues that did not warrant exclusion in 37/304 (13%) children (allergies, asthma, eczema, or febrile seizures). Consequently, cohort 1 included 304 children, evenly split between males (N = 152) and females (N = 152). All children from cohort 1 were assessed at a single time point.

#### Cohort 2

A total of 98 primary caregivers provided consent and completed the sociodemographic/medical history form. One child was excluded based on sensorimotor barriers to assessment and another was excluded due to an incomplete assessment. No children were excluded based on neurologic disease/illness/injury. Mild medical issues that did not warrant exclusion were endorsed by caregivers for 14/96 (15%) children (e.g., allergies, asthma, eczema, or febrile seizures). Consequently, cohort 2 included 96 children (38 males, 58 females). All children from cohort 2 were evaluated at two time points, spaced 2 weeks apart, to assess test-retest reliability. Additionally, a subset of 12 caregivers provided consent for their children to be videotaped, which facilitated the evaluation of inter-rater agreement.

There were no differences between cohorts 1 and 2 in caregiver income bracket, χ^2^ (4) = 1.55, *p* = .817, *V* = .06, or marital status, χ^2^ (5) = 7.97, *p* = .158, *V* = .14, or in the proportion of children with mild medical concerns, χ^2^ (1) = .42, *p* = .517, *V* = .03. Education level differences were marginal, χ^2^ (2) = 6.00, *p* = .049, *V* = .12. The two cohorts were combined into one sample to investigate subtest score distributions, associations with demographic variables, and construct validity. Characteristics of the combined sample of 400 children, ages 3–5 years, are presented in Table 2.

### GLAMS subtest score distributions

On the SLT, seven children (2%) showed zero learning and these scores were removed from further analyses, along with their corresponding delayed recall and recognition scores. Among the remaining children, 120 (30%) demonstrated some learning across trials (an increase in performance across two subsequent trials) and 273 (68%) showed a positive learning curve. Total learning scores were normally distributed with acceptable skewness (−.19) and kurtosis (−.64). Delayed recall scores approached normality with acceptable skewness (−.06) but marginally negative kurtosis (−.97), indicative of a platykurtic distribution with a low peak, thin tails, and moderately spread-out values around the mean. Recognition discriminability displayed acceptable skewness (.71) and a marginally platykurtic distribution (−.94), with a large number of zero values (38%). A recognition discriminability score of zero indicates a positive response bias (saying “yes” to every item), which was further explored across age groups. There was a clear tendency for younger children to exhibit this positive response bias, with 63% of 3-year-olds and 36% of 4-year-olds responding “yes” to every recognition item, compared to 12% of 5-year-olds.

On the FNBT learning trials, 64 children (15%) demonstrated zero learning, and these scores (along with corresponding delayed recall scores) were excluded from further analyses. Among the remaining children, 90 (23%) showed some learning across trials, while 246 (62%) displayed a positive learning curve. Total learning scores were non-normally distributed due to positive skew (1.08) and marginal kurtosis (0.95). Delayed recall scores were also non-normally distributed due to positive skew (1.16) and excess kurtosis (1.83), with high peak concentration around the median of 1 (mode = 1). Given results from these descriptive analyses, statistical tests that assume normality were run on SLT scores, and nonparametric tests were utilized for FNTB scores.

### Sociodemographic effects

#### Age

As age increased across 6-month age brackets, a higher proportion of children showed a positive learning curve on SLT total learning, χ^2^ (10) = 34.66, *p* < .001, *V* = .21, and FNBT total learning, χ^2^ (10) = 31.76, *p* < .001, *V* = .20.

Moderate age effects were observed on SLT total learning, *r*(393) = .53, *p* < .001, delayed recall, *r*(392) = .51, *p* < .001, and recognition discriminability, *r*(392) = .48, *p* < .001, subtests, indicative of performance improvement with age. There were no age effects on SLT error scores, (e.g., intrusions and repetitions), with the exception of small age effects on recognition test false positive errors, *r*(336) = −.32, *p* < .001. Within the FNBT, small age effects were observed on FNBT learning, ρ(336) = .23, *p* < .001 and delayed recall, ρ(336) = .23, *p* < .001, also indicative of performance improvement with age.

#### Sex effects

A small sex effect was observed on SLT total learning, *t (391)* = −2.57, *p* < .01, *d* = 0.26, and recognition discriminability, *t (390)* = −1.78, *p* < .05, *d* = 0.18, indicative of a female performance advantage. A moderate sex effect was found on SLT delayed recall, *t (390)* = −3.17, *p* < .01, *d* = 0.58. On average, girls recalled 0.5 more words than boys. However, no sex effects were noted on SLT error scores, FNBT total learning, *U* = 13,444, *p* = .488, or FNBT delayed recall, *U* = 14,124, *p* = .985.

#### Caregiver income and education

There were small effects of income on SLT recognition discriminability, ρ(345) = .12, *p* = .029; a higher level of income was associated with a performance advantage. No other GLAMS subtests were correlated with caregiver income or education level.

### Reliability

Internal consistency was acceptable among all nine GLAMS subtest trials (α = .74) and among the five SLT trials (α = .72) and four FNBT trials (α = .74). In an exploratory factor analysis with direct oblimin rotation, KMO was sufficient (0.71), and Bartlett’s test was significant (*p* < .001). The analysis revealed two latent factors, with Factor 1 contributing 50% and Factor 2 an additional 22% to the total variance prior to rotation. Rotated sums of squared loadings are provided in Table 3. Within the rotated component matrix, three subtests exhibited high loadings on Factor 1 (SLT learning, delayed recall, and recognition discriminability), while two subtests displayed high loadings on Factor 2 (FNBT total learning and delayed recall). These results support the GLAMS as a measure of a unified construct (memory), with two latent factors (verbal and visual-verbal associative memory) accounting for 72% of the variance.

The test-retest interval duration was 2 weeks, with a median of 14 days across all three settings (school, home, clinic). Acceptable test-retest reliability was observed for SLT total learning, SLT recognition discriminability, and FNBT total learning (Table 4). Relatively smaller (but still significant) reliability coefficients were observed for SLT delayed recall and FNBT delayed recall (Table 4). Higher reliability coefficients for total learning scores are consistent with classical test theory predictions of higher reliability for multiple-item measures (Cappelleri et al., 2014). The relatively lower delayed recall reliability coefficients should be interpreted in the context of the rapid developmental gains achieved during early childhood. Test-retest reliability coefficients remained acceptable across test settings (home, school, clinic) for SLT total learning and delayed recall but were more variable for FNBT subtests (Table 4), with higher reliability coefficients obtained in the clinic, relative to home, for FNBT total learning.

### Inter-rater reliability

Inter-rater agreement was evaluated across four raters who independently observed and scored videos of 12 GLAMS administrations to 3-, 4-, and 5-year-old children (four in each age group). Substantial to perfect agreement was achieved on all trials (Table 5). Slightly lower agreement was observed in ratings of “repeats” and “intrusions” due to ambiguity in coding of repeated intrusions. This prompted clarification of scoring criteria to specify that a repeated intrusion should be counted as an “intrusion.”

### Construct validity

There was a positive correlation between NEPSY-II Sentence Repetition and SLT total learning, *r (388)* = .31, *p* < .001, delayed recall, *r (387)* = .27, *p* < .001, and recognition discrimination, *r (387)* = .26, *p* < .001, as well as with FNBT total learning, ρ(335) = .28, *p* < .001, and delayed recall, ρ(336) = .21, *p* < .01. These findings support convergent validity between a measure of verbal working memory and all five core GLAMS subtests. Furthermore, the absence of a positive correlation between NEPSY-II visuomotor precision and any of the GLAMS subtests supports divergent validity with the purportedly orthogonal construct of speeded hand-eye coordination.

Further exploration with the NEPSY-II block construction subtest revealed small positive correlations with FNBT total learning, ρ(335) = .14, *p* < .01, and delayed recall, ρ(336) = .19, *p* < .001, but not with other GLAMS subtests. This aligns with the expected visuospatial demands of face-name associative memory. However, correlations with the NEPSY-II statue subtest yielded unexpected positive association with SLT total learning, *r (389)* = .31, *p* < .001, delayed recall, *r (389)* = .29, *p* < .001, and recognition discrimination, *r (389)* = .26, *p* < .001, as well as with FNBT total learning, ρ(335) = .15, *p* < .01. This highlights inhibitory control as a significant component of learning and memory performance and underscores the inherent executive demands of standardized testing in young children.

### Normative data

#### Shopping List Test (SLT)

Means and standard deviations for SLT scores are provided in Table 6 to facilitate individual-level normative adjustments. Due to the positive relationship between age and subtest scores, the sample was stratified into 6-month age blocks for age adjustment. Given that sex effects were observed on SLT total learning, delayed recall, and recognition discriminability subtests, the sample was further stratified by sex to facilitate age- and sex-based adjustments for these subtests. The data, along with an Excel normative score calculator, can be downloaded from the following site (www.cccnd.org).

#### Face-Name Binding Test (FNBT)

Given the non-normal distribution of scores on FNBT total learning and delayed recall subtests, cumulative percentages were obtained for scores across 6-month age blocks (Table 7).

## Discussion

This study presents psychometric and normative data for the GLAMS in 400 preschool children. Overall, children effectively engaged with GLAMS subtests. Results show acceptable internal consistency, test-retest reliability, and inter-rater reliability. The construct validity of GLAMS as a domain-specific memory measure is supported by a positive (convergent) relationship with a gold standard measure of working memory from the NEPSY-II. Principal components analysis reveals a two-factor structure that aligns with the theoretical constructs of verbal and visual-verbal associative memory. These findings offer promising preliminary support for the use of the GLAMS Grenadian preschool children across research and clinical settings. However, further work is needed to enhance confidence in construct validity. It is important to interpret results with consideration of each subtest’s psychometric properties across different demographic sub-groups, as discussed in more detail below.

As expected, a positive relationship with age was evident across all GLAMS subtests, highlighting the need for age adjustment. Notably, a higher percentage of children demonstrated sufficient learning across trials on the SLT (98%) compared to the FNBT (85%), although learning improved with age for both tests. SLT scores more closely approximated a normal distribution, with sensitivity at the extremes of the sampling distribution. In contrast, FNBT exhibited floor effects, which suggests that it was challenging for preschool children. Thus, FNBT can be used to identify children who are advanced for their age but not those who are delayed. On the other hand, SLT learning and delayed recall subtests can be considered psychometrically robust for detecting both delayed and advanced maturation. Whether delayed performance signals pathology in hippocampal-cortical memory networks remains to be demonstrated in future studies.

The shopping list length of four words (for 3-year-olds) and six words (for 4- and 5-years-olds) is much shorter than pediatric list-learning tests developed in HICs (Goodman et al., 1999; Kasperek et al., 2023). For instance, the 15-item CVLT-C was used in a sample of 4-year-olds from the United States. Although performance characteristics suggested a positive learning curve, concerns with floor effects were raised (Goodman et al., 1999). By using shorter lists in preschool children, we were able to eliminate such floor effects. This underscores the importance of co-developing measures with community stakeholders to improve sensitivity at the tails of the distribution in younger children.

The psychometric properties of the SLT total learning and delayed recall trials were satisfactory. However, there was evidence of a positive response bias in the “yes/no” recognition testing for 63% of 3-year-olds and 36% of 4-year-olds in the sample. This is consistent with prior studies on recognition memory performance profiles in 4-year-old children (Goodman et al., 1999). By 5 years of age, this positive response bias considerably diminished to 12%. A similar age-associated decrease in positive response bias during the preschool years has been observed cross-culturally (Okanda et al., 2012). Given this concordance, we recommend deferring administration of the recognition trial until children reach the age of five.

Interestingly, we observed a female advantage in list learning and delayed recall, which is a robustly replicated finding in older children (Brooking et al., 2012; Kramer et al., 1997; Lowe et al., 2003) and adults (Pauls et al., 2013; Sundermann et al., 2019) but has not been previously demonstrated in children as young as 3 years of age. From a neurodevelopmental perspective, this raises the possibility of a very early emergence of the female learning advantage, which should be further explored and replicated in future studies.

There were several findings that support future scale-up of the GLAMS SLT to other LMICs. First, we found acceptable test-retest reliability coefficients across diverse settings (home, school, clinics). Data were often collected in sub-optimal conditions. Our demonstration of reliability across these settings is encouraging, considering the real-world assessment challenges typically encountered in limited resource settings (Sabanathan et al., 2015). Second, the median education (secondary school) and monthly income ($1001–$2000 XCD) of caregivers in our sample is consistent with World Bank data for Grenada, which supports population-level generalizability. Third, the GLAMS SLT can be administered by trained assessors with bachelor’s-level education. In our setting, TECAs were from the Caribbean and able to engage culturally and linguistically with the children. This is important to note because the GLAMS is an interactive measure. It involves eye contact, encouragement, and redirection of attention. We suspect these to be important components for sustained child engagement in the preschool years. For research purposes, this means that budgets should consider hiring college-level study personnel and training costs, as sufficient training to meet performance criteria and maintenance of quality assurance is essential to the collection of reliable data. Fourth, the SLT is available free of charge. It can be downloaded from the Caribbean Center for Child Neurodevelopment website (www.cccnd.org), along with a scoring calculator to generate age- and sex-adjusted scores.

Given its robust psychometric properties, low cost, and ease of scalability, the GLAMS SLT can be considered for use in the English-speaking Caribbean as a performance-based measurement tool in conjunction with the Early Childhood Development Index 2030 (ECDI2030). The ECDI2030 is a 20-item caregiver questionnaire designed to assess the achievement of key developmental milestones in children between the ages of 24 and 59 months (United Nations Children’s Fund [UNICEF], 2023). It is the official measure for monitoring the achievement of United Nations Sustainable Development Goals (https://unstats.un.org/sdgs), indicator 4.2.1 (proportion of children developmentally on track). By pairing the GLAMS with a globally recognized and validated developmental indicator, researchers can track domain-specific memory performance in conjunction with domain-general developmental achievements. Further translation, adaptation, and scale-up of the GLAMS could extend this capacity to other French- and Spanish-speaking Caribbean islands, as well as beyond the Caribbean.

### Limitations

We found preliminary support for the construct validity of the GLAMS as a memory measure of verbal and visual-verbal associative memory; however, confirmatory factor analysis is recommended in future independent samples to validate the two-factor structure. More work is needed to assess whether modifications to the FNBT could improve its performance in young children. Face perception develops rapidly during the preschool years (Bruce et al., 2000), with some studies showing adult-like processing of faces as young as 4 years of age (de Heering et al., 2007). However, little is known about the extent to which children in this age range can reliably associate faces with names. Future inclusion of face discrimination and multiple-choice recognition trials in the FNBT may shed light on whether performance is limited by the face recognition or visual-verbal associative memory demands of this task.

We did not examine concurrent or predictive validity against external criteria (e.g., cognitive, behavioral, and social functioning at school entry; development of neurodevelopmental disorders, etc.). It remains unclear whether the GLAMS is sensitive to clinical pathology in hippocampal-cortical memory networks, change over time, or response to interventions. Finally, the use of NEPSY-II Sentence Repetition as a convergent measure of immediate memory was not ideal, in that it arguably relies on frontal-parietal systems supporting verbal working memory (Thaler et al., 2013). However, the interaction between working memory and associative memory systems during the formation of hippocampal-based episodic memory representations is supported by Baddeley’s multicomponent model of working memory (Repovs & Baddeley, 2006). The degree to which this applies to episodic memory network development in preschool children remains unclear due to a paucity of research on this topic. These limitations suggest promising next steps for further validation of the GLAMS. The development of alternate forms is needed to reduce practice/learning effects in longitudinal studies. Ongoing work is underway to validate the GLAMS in older children, with the inclusion of a “T8” version to raise the test ceiling for 5- to 10-year-old children. Although confirmatory factor analysis, validation against clinical groups and additional measures of episodic memory, and extension into older children are needed, the robust psychometric properties of the GLAMS highlight its promise for future lifespan brain health investigations in LMICs.

Finally, there may be concerns about developing a new memory measure in Caribbean preschool children, rather than adapting existing tools such as the NIH Early Childhood Toolbox (Denboer et al., 2014). Existing measures come with a wealth of existing psychometric and normative data. However, most existing measures (and the psychometric/normative data associated with them) were developed in HICs, without considering the cultural, economic, and logistical challenges of assessment in limited resource environments. For example, the NIH Early Childhood Toolbox requires costly computer hardware that is cumbersome to carry into the field for research. Children in LMICs may have less exposure to computers at home, which can place them at a disadvantage relative to children in HICs. Once validated, neuropsychological tests should have a pathway into the clinic. Test costs can serve as a barrier to adoption into public healthcare settings, which worsens disparities in care between public and private health systems. We argue for a shift in the neuropsychological landscape toward incorporating assessment solutions that have been generated from LMICs and may be of broader benefit to limited resource settings globally.

## Figures and Tables

**Table 1.** Neuropsychological test battery

Subtest name	Order	Description	Response criterion

GLAMS SLT learning	1	Verbal learning; three learning trials; oral-auditory stimulus presentation; no feedback provided	Accuracy: 1 point for each correct item
GLAMS FNBT learning	2	Visual-verbal associative learning; three learning trials; visual presentation of faces; auditory presentation of names; feedback provided	Accuracy: 1 point for each correct item
NEPSY-II block construction	3	Visuospatial and visuomotor ability; timed; child is asked to reproduce three-dimensional block constructions from models or from two-dimensional drawings.	Accuracy: 1 point for each correct item. Response Time: Bonus points for fast completion.
NEPSY-II statue	4	Motor persistence and inhibition; child is asked to maintain a body position with eyes closed during a 75-second period and to inhibit the impulse to respond to sound distracters.	Accuracy: 2 points for no errors (body movements, eye openings vocalizations) or 1 point for one error, during a 5-second interval
NEPSY-II visuomotor precision	5	Graphomotor skills; timed; child uses his or her preferred hand to draw lines inside of tracks as quickly as possible.	Accuracy: Errors scored for line deviations and task incompletion Response time: Quickness to complete
GLAMS SLT delayed recall	6	Verbal memory retrieval; free recall of shop list items after a 20-minute delay	Accuracy: 1 point for each correct item
GLAMS SLT recognition discriminability	7	Verbal memory recognition; target items presented among semantically similar (FS) and different (FD) foils	Accuracy: 1.5 points for correct hit; −0.5 points for incorrect FS; −1 point for incorrect FD
GLAMS FNBT delayed recall	8	Visual-verbal associative memory; free recall of names that were matched to faces after a 20-minute delay	Accuracy: 1 point for each correct item
NEPSY-II Sentence Repetition	9	Verbal working memory; child is asked to repeat sentences of increasing complexity and length.	Accuracy: 2 points for no errors (omitting, changing, adding or transposing words) or 1 point for 1–2 errors

*Note.* GLAMS = Grenada Learning and Memory Scale, SLT = Shopping List Test, FNBT = Face-Name Binding Test.

**Table 2.** Demographic characteristics of the sample (N = 400)

Child characteristics	

Biological sex at birth n (%)	
Males	190 (48%)
Females	210 (52%)
Age (in months)	
Mean (SD)	52.73 (9.69)
Median	53
Mode	50
Range	36–71
Caregiver characteristics	
Education n (%)	
Primary	57 (14)
Secondary (median)	203 (51)
Tertiary or higher	132 (33)
Missing	8 (2)
Marital status n (%)	
Married	90 (23)
Widowed	6 (2)
Divorced	5 (1)
Separated	10 (3)
Partnered	61 (15)
Single	210 (52)
Missing	18 (4)
Monthly income (Eastern Caribbean dollars) n (%)	
<500	33 (11)
500–1000	96 (32)
1001–2000 (median)	48 (16)
2001–3000	35 (11)
>3000	55 (18)
Missing	37 (12)

**Table 3.** Results from exploratory principal components analysis with varimax rotation

Total variance explained						

	Initial eigenvalues			Rotated sum of squared loadings		
Component	Total	% of Variance	Cumulative %	Total	% of Variance	Cumulative %

1	2.49	49.99	49.99	2.50	49.99	49.99
2	1.09	21.75	71.74	1.09	21.75	71.74
3	0.59	11.79	83.54			
4	0.43	8.65	92.18			
5	0.39	7.82	100.00			

Rotated component pattern matrix						

			Principal components			
Subtest			Component 1		Component 2	

SLT total learning			0.82		0.30	
SLT delayed lecall			0.85		0.33	
SLT recognition discriminability			0.77		0.30	
FNBT total learning			0.34		0.90	
FNBT delayed recall			0.35		0.89	

*Note*. SLT = Shopping List Test; FNBT = Face-Name Binding Test.

**Table 4.** Test-retest reliability

Subtest	*r*		95% CI		*p*	

SLT total learning	.72		[0.61, 0.80]		<.001	
SLT delayedrecall	.56		[0.41, 0.69]		<.001	
SLT recognition discriminability	.80		[0.72, 0.86]		<.001	
FNBT total learning	.62		[0.48, 0.74]		<.001	
FNBT delayed recall	.45		[0.27, 0.59]		<.001	

Test-retest reliability across settings						

	Home (*n* = 30)		School (*n* = 33)		Clinic (*n* = 33)	
	*r*	*p*	*r*	*p*	*r*	*p*

SLT total learning	.69	<.001	.66	<.001	.78	<.001
SLT delayed recall	.63	<.001	.52	<.002	.54	<.001
SLT recognition discriminability	.87	<.001	.92	<.001	.65	<.001
FNBT total learning	.37	<.05	.49	<.01	.80	<.001
FNBT delayed recall	.51	<.01	.26	.142	.63	<.001

*Note. r* = Pearson’s *r*, CI = confidence interval, SLT = Shopping List Test; FNBT = Face-Name Binding Test.

**Table 5.** Inter-rater agreement across all GLAMS trials (4 raters, 12 videos)

Variable	κ	Qualitative labeling of effect size	95% CI	*p*

SLT learning trial 1	1.00	Perfect agreement	[0.89, 1.11]	<.001
SLT learning trial 2	.88	Almost perfect agreement	[0.75, 0.99]	<.001
SLT learning trial 3	1.00	Perfect agreement	[0.88, 1.13]	<.001
SLT delayed recall	.76	Substantial agreement	[0.65, 0.87]	<.001
SLT test repeats	.65	Substantial agreement	[0.48, 0.82]	<.001
SLT intrusions	.70	Substantial agreement	[0.58, 0.83]	<.001
SLT recognition hits	.93	Almost perfect agreement	[0.77, 1.09]	<.001
SLT similar foils	.92	Almost perfect agreement	[0.76, 1.07]	<.001
SLT different foils	.91	Almost perfect agreement	[0.72, 1.09]	<.001
SLT recognition discriminability	.88	Almost perfect agreement	[0.77, 0.99]	<.001
FNBT learning trial 1	.88	Almost perfect agreement	[0.75, 0.99]	<.001
FNBT learning trial 2	.87	Almost perfect agreement	[0.75, 0.99]	<.001
FNBT learning trial 3	1.00	Perfect agreement	[0.85, 1.15]	<.001
FNBT delayed recall	1.00	Perfect agreement	[0.87, 1.13]	<.001

*Note.* κ = Fleiss’ kappa, CI = confidence interval, SLT = Shopping List Test; FNBT = Face-Name Binding Test.

**Table 6.** Shopping List Test means and variance across 6-month age blocks

Shopping List Test learning, delayed recall, and error scores									

		Total learning		Delayed recall		Repetitions		Intrusions	
Age (months)	N	Mean	SD	Mean	SD	Mean	SD	Mean	SD

36–42	79	6.39	2.63	1.33	1.21	0.35	0.82	1.24	1.50
43–48	56	7.8	2.85	1.95	1.27	1.29	2.95	1.25	1.71
49–54	75	9.48	2.74	2.52	1.58	0.73	1.18	2.24	3.19
55–60	85	10.29	3.08	3.20	1.56	0.55	0.78	0.59	1.10
61–66	61	11.39	2.90	3.49	1.39	0.59	1.10	1.72	2.03
67–72	37	11.27	2.30	3.86	1.25	0.68	1.55	1.97	2.44

Shopping List Test yes/no recognition discriminability, hits, and false positives									

		Recognition discriminability		Total hits		Semantically similar foils		Semantically different foils	
Age (months)	N	Mean	SD	Mean	SD	Mean	SD	Mean	SD

36–42	79	0.64	1.46	5.83	1.23	1.75	0.58	3.44	1.37
43–48	56	0.67	1.72	6.30	2.01	1.95	0.78	3.68	1.76
49–54	75	2.30	3.41	7.82	1.99	1.88	1.25	3.64	2.57
55–60	85	3.48	3.76	7.41	2.46	1.41	1.26	2.53	2.59
61–66	61	4.05	3.57	7.23	2.29	1.16	1.08	1.95	2.25
67–72	37	6.05	3.50	8.07	1.51	0.80	1.10	1.22	2.15

**Table 7.** Face-Name Binding Test cumulative percentages across 6-month age blocks

Cumulative percentages for Face-Name Binding Test total learning											

Age (months)	N	0–9th	10–19th	20–29th	30–39th	40–49th	50–59th	60–69th	70–79th	80–89th	90–99th

36–42	60	–	–	1	–	–	2	3	4	5	6+
43–48	44	–	–	1	–	2	–	3	–	4–5	6+
49–54	64	–	–	1	–	–	2	3	–	4–6	7+
55–60	77	–	1	–	2	3	–	4	5	6	7+
61–66	56	–	–	1	2	3	–	4	5	6–7	8+
67–72	35	1	–	2	3	–	4	–	5	6–7	8+

Cumulative percentages for Face-Name Binding Test delayed recall											
		
Age (months)	N	0–9th	10–19th	20–29th	30–39th	40–49th	50–59th	60–69th	70–79th	80–89th	90–99th

36–42	60	–	–	–	–	–	0	–	–	1	2+
43–48	44	–	–	–	0	–	–	–	1	–	2+
49–54	64	–	–	–	0	–	–	–	1	–	2+
55–60	77	–	–	0	–	–	–	1	–	2	3+
61–66	56	–	–	0	–	–	1	–	–	2	3+
67–72	35	–	–	0	–	–	1	–	–	2	3+

**Figure A1.**
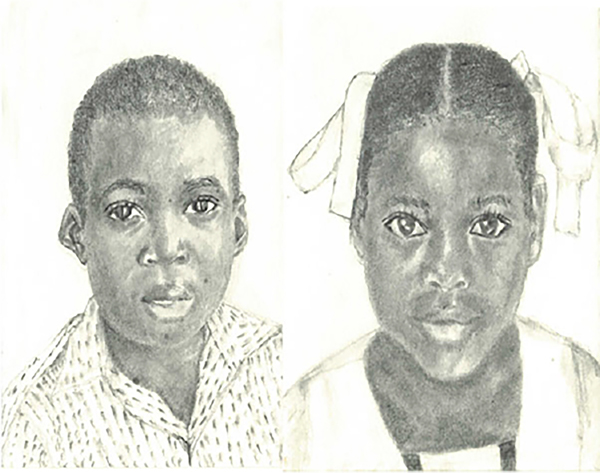
Sample stimuli from the Face-Name Binding Test (FNBT).

## APPENDIX A

### Community stakeholder involvement in GLAMS development process

The GLAMS was developed through an iterative process, with input and involvement from Grenadian stakeholders at various stages of the process. Stakeholders included members of the study team from Grenada, Trinidad, and the Bahamas, graduate students from the Masters in Community and Clinical Psychology Program (MACCP) at St George’s University in Grenada, and Grenadian healthcare workers, early childhood educators, and parents. We held two focus groups, one during initial test development and one after pilot data had been collected. The first focus group involved eight MAACP students; the second involved early childhood coordinators from Ministries of Education across the Caribbean during a workshop on early childhood assessment and interventions, hosted in Grenada by the UNICEF – Eastern Caribbean Office. Input from nurses and healthcare workers was informally sought during pilot testing.

The Shopping List Test (SLT) was modeled after the California Verbal Learning Test – Children’s Version. Focus group participants communicated that a trip to the shop with siblings or parents was a common occurrence for preschool children in Grenada, particularly in rural village settings. Consensus was reached on shopping list items to include as targets and foils, based on items familiar to the region. Participants felt that the instructions and 8-item target word list were too long for preschool children. Suggestions were made to simplify the instructions and reduce the word list to six items. The word list was further reduced to four items for 3-year-olds based on iterative feedback from trained early childhood assessors (TECAs) during pilot testing, whereas six items were retained for children 4 and 5 years of age.

The Face-Name Binding Test (FNBT) was modeled after the Memory for Names subtest from the NEPSY-II due to initial focus group consensus that face-name association was an ecologically familiar activity among preschool children in Grenada. A Grenadian study team member who was also a visual artist drew pictures of children’s faces that resembled children from the region (Figure A1). Study team members generated a list of names that were considered common in the region. The number of items presented to different age groups were purposely aligned with the SLT. These stimuli were trialed during pilot testing and positively received by parents and healthcare workers. After pilot testing was complete, we demonstrated the full test to a group of early educators (UNICEF-Eastern Caribbean Office workshop), who provided positive feedback on the cultural acceptability of test items.

When designing the overall cognitive test protocol, we selected NEPSY-II subtests to assess the convergent and divergent validity of GLAMS subtests. Given the limited attention spans of preschool children, we prioritized subtests that were quick, simple to administer across various contexts (clinics, schools, homes) and considered culturally appropriate by Grenadian members of the study team. To assess convergent validity, we considered all of the preschool memory domain subtests from the NEPSY-II. There were concerns raised about the cultural familiarity and administration time/burden of the Narrative Memory and Memory for Designs subtests. Ultimately, Sentence Repetition was selected. Although it may be considered a measure of verbal working memory, it presumably recruits overlapping substrates for episodic memory formation. According to Baddeley’s multicomponent model of working memory (Repovs & Baddeley, 2006), tasks such as Sentence Repetition probe the “episodic buffer,” which is a temporarily limited capacity storage system responsible for integrating the material into longer-term episodic representations. The length, complexity, and rehearsal parameters needed for a short-term trace to become a long-term representation in preschool children remains unclear, but it can be presumed that Sentence Repetition shares substrates with episodic memory based on strong cross-correlation (*r* = 0.42) with the Narrative Memory Freed and Cued Recall Score in children 3–4 years of age from the NEPSY-II Clinical and Interpretive Manual (Korkman et al., 2007).

## APPENDIX B

### Behavioral observations form

As mentioned in the manuscript text, TECAs were trained to observe children’s behavior for signs of fatigue, disengagement, frustration, and distractibility, and to intervene with attempts to recapture attention, eliminate distractions, downregulate frustration, or reschedule the assessment if necessary. As the study progressed, TECAs were instructed to note their behavioral observations on a form. This was encouraged to help the team identify common behavioral sources of low performance (“zero scores”) on learning measures. These behavioral observations were then incorporated into a checklist at the end of cohort 1 data collection. This checklist was formally included at the end of the record form for cohort 2 data collection to prompt TECAs to indicate if any behavioral concerns were present during testing. Given that the checklist was not available from study onset, formal results were not incorporated into the study manuscript. However, the checklist was utilized for all children in cohort 2, and the results are summarized in Tables C1 and C2.

**Table C1.** Behavioral observations of cohort 2 (*N* = 96)

Behavior checklist	Yes *n* (%)	No *n* (%)	Missing *n* (%)

Is the child engaged?	88 (91.7)	1 (1.1)	7 (7.3)
Is the child cooperative?	88 (91.7)	1 (1.1)	7 (7.3)
Is the child distractible?	21 (21.9)	68 (70.8)	7 (7.3)
Is the child hungry/tired? (adult report/cues from child)	5 (5.2)	84 (87.5)	7 (7.3)
Does the child exhibit performance anxiety?	4 (4.2)	85 (88.5)	7 (7.3)
Does the child exhibit comprehension of instructions?	88 (91.7)	1 (1.1)	7 (7.3)
Does the child produce intrusions?	21 (21.9)	68 (70.8)	7 (7.3)
Does the child echo back the last thing you said?	2 (2.1)	87 (90.6)	7 (7.3)
Assessor Error/Parent (Caregiver) Interference	7 (7.3)	82 (85.4)	7 (7.3)
Is the testing room a conducive environment?	79 (82.3)	6 (6.3)	11 (11.5)

**Table C2.** Environmental testing conditions of cohort 2 (*N* = 96)

Environmental conditions	Low *n* (%)	Moderate *n* (%)	High *n* (%)	Missing *n* (%)

1. Noise level	66 (68.8)	21 (21.9)	2 (2.1)	7 (7.3)
2. Lighting	0 (0.0)	14 (14.6)	75 (78.1)	7 (7.3)
3. Distractions	60 (62.5)	27 (28.1)	2 (2.1)	7 (7.3)

### GLAMS ASSESSMENT CHECKLIST

**Child ID:**____________ **Date:** ______________

**Assessor:**__________________

**1.** Behavioral observations

Behavior checklist	Yes	No

*Is the child engaged?*		
*Is the child cooperative?*		
*Is the child distractible?*		
*Is the child hungry/tired? (adult report/cues from child)*		
*Does the child exhibit performance anxiety?*		
*Does the child exhibit comprehension of instructions?*		
*Does the child produce intrusions?*		
*Does the child echo back the last thing you said?*		
*Assessor Error/Parent (Caregiver) Interference*		

Any additional notes:

______________________________________________

______________________________________________

**2.** Select Testing Environment: Home School Clinic

Environmental conditions	Low	Moderate	High

1. Noise level			
2. Lighting			
3. Distractions			

Is the testing room a conducive environment? Yes/no Any additional notes:

______________________________________________

______________________________________________
